# Transitory masculinities in the context of being sick with prostate cancer*

**DOI:** 10.1590/1518-8345.3248.3224

**Published:** 2019-12-05

**Authors:** Jeferson Santos Araújo, Vander Monteiro da Conceição, Marcia Maria Fontão Zago

**Affiliations:** 1 Universidade Federal do Sul e Sudeste do Pará, Faculdade de Saúde Coletiva, Marabá, PA, Brazil.; 2 Universidade Federal da Fronteira Sul, Curso de Graduação em Enfermagem, Chapecó, SC, Brazil.; 3 Universidade de São Paulo, Escola de Enfermagem de Ribeirão Preto, PAHO/WHO Collaborating Centre for Nursing Research Development, Ribeirão Preto, SP, Brazil.

**Keywords:** Anthropology Medical, Prostatic Neoplasms, Masculinity, Men’s Health, Oncology Nursing, Qualitative Research, Antropologia Médica, Neoplasias da Próstata, Masculinidade, Saúde do Homem, Enfermagem Oncológica, Pesquisa Qualitativa, Antropología Médica, Neoplasias de la Próstata, Masculinidad, Salud del Hombre, Enfermería Oncológica, Investigación Cualitativa

## Abstract

**Objective:**

to interpret the meanings attributed by men with prostate cancer to the experience regarding their bodies and masculinities during illness.

**Method:**

ethnographic research with 17 men, guided by the narrative method and theoretical framework of medical anthropology and masculinities. The information was collected through recorded interviews, direct observation and field diary records, which were analyzed by inductive thematic analysis.

**Results:**

men undergo body and identity transformations when they get sick with prostate cancer, transiting through multiple masculinities, resigning their actions, and occupying subordinate positions in relation to other healthy bodies, which are marginalized in their social relationships and allied with regard to establishing their affective relationships.

**Conclusion:**

this evidence enhances and deepens the knowledge disclosed in the literature and contributes to the strengthening of nursing care actions when dealing with the sick.

## Abstract (pt)

**Objetivo:**

interpretar os significados atribuídos à experiência dos homens com o câncer de próstata sobre seus corpos e suas masculinidades durante o adoecimento, com base na concepção da antropologia médica e das masculinidades.

**Método:**

pesquisa etnográfica norteada pelo método narrativo e pelos referenciais teóricos da antropologia médica e das masculinidades com 17 homens. As informações foram colhidas por meio de entrevistas gravadas, observação direta e registros em diário de campo, os quais foram analisados pela análise temática indutiva.

**Resultados:**

os homens sofrem transformações corporais e identitárias durante o adoecimento pelo câncer de próstata, transitando por múltiplas masculinidades, resignando suas ações, ocupando posições subordinadas para com outros corpos sadios, marginalizadas em suas relações sociais e cúmplices no estabelecimento de suas relações afetivas.

**Conclusão:**

estas evidências potencializam e aprofundam os conhecimentos divulgados na literatura e contribuem para o fortalecimento de ações de cuidado de enfermagem ao lidar com os adoecidos.

## Abstract (es)

**Objetivo:**

interpretar los significados atribuidos a la experiencia de los hombres con el cáncer de próstata sobre sus cuerpos y sus masculinidades durante la enfermedad.

**Método:**

investigación etnográfica orientada por el método narrativo y por los referenciales teóricos de la antropología médica y de las masculinidades con 17 hombres. Las informaciones fueron recogidas por medio de encuestas grabadas, observación directa y registros en diario de campo, los cuales fueron analizados por el análisis temático inductivo.

**Resultados:**

los hombres sufren transformaciones corporales y de identidad durante la enfermedad por el cáncer de próstata, transitando por múltiples masculinidades, resignando sus acciones, ocupando posiciones subordinadas para con otros cuerpos sanos, marginalizadas en sus relaciones sociales y cómplices en el establecimiento de sus relaciones afectivas.

**Conclusión:**

estas evidencias potencian y profundizan los conocimientos divulgados en la literatura y contribuyen para el fortalecimiento de acciones de cuidado de enfermería al lidiar con los enfermados.

## Introduction

According to US statistics^(^ 1 ^)^, one in six men in the world will be diagnosed with prostate cancer (PC) during their lifetime. Regardless of population color and ethnicity, the disease still prevails as a silent threat to the Latin men’s health as the third cause of death from noncommunicable - or chronic - diseases^(^ 1 ^)^. For the biennium 2018-2019, there are estimated 66,120 new cases per 100,000 Brazilian men, and the incidence is six times higher in developed countries, such as the United States, Canada, and England^(^ 2 ^)^.

The cure for PC is a phenomenon investigated from multiple scientific perspectives and each year new discoveries are evidenced. Parallel to this pursuit, the experience of surviving or living with the disease is still a field to be strengthened for the improvement of new techniques of care and implementation of public policies. Researchers^(^ 3 ^)^ recognize that treatments for PC, except for vigilance, usually result in unpleasant side effects that end up leaving men vulnerable to aspects that involve their masculinity. These effects include sexual dysfunction, changes in body image and social stigma experienced by almost all those submitted to prostatectomy^(^ 3 ^-^ 4 ^)^. 

Faced with male vulnerability resulting from the treatments, men undergo transformations in their bodies and social identities during their lifetime. As much as the transition is a natural process for humankind, being ill intensifies this phenomenon and provides new experiences in the way they face the disease^(^ 5 ^)^. Thus, understanding the male transitions that occur due to PC is extremely relevant to strengthening treatments, education and research directed at this population.

The scientific literature has evidenced gaps regarding the knowledge of the subject. To illustrate this question, a qualitative meta-synthesis^(^ 6 ^)^ was performed on the following databases: Latin American and Caribbean Literature - LILACS, Medical Literature Analysis and Retrieval System Online - MEDLINE, and Cumulative Index for Nursing and Allied Health Literature - CINAHL, according to PRISMA recommendations, using DeCS and MeSH scientific descriptors *masculinity* and *prostatic neoplasms*, guided by the Boolean operator *and*. The filters used were human studies, with qualitative methodology, in English, Spanish, and Portuguese. After finding and reading the titles, abstracts and results, the data were extracted and synthesized by two different researchers, who evidenced that among the 21 references selected, discussions about men’s health issues related to sexuality, diet, prevention, diagnosis and treatment prevailed, but the transitions occurring in the body and masculinities during the disease were not included in this context.

Therefore, the relevance of this research is justified based on need to know how man transitions his body and masculinities when he gets sick with PC. For the purpose of acquiring this knowledge, it was sought to interpret the meanings attributed by men with prostate cancer to the experience regarding their bodies and masculinities during illness.

## Method

This is an ethnographic research guided by the narrative method and theoretical framework of medical anthropology^(^ 7 ^)^ and masculinities^(^ 8 ^)^. 

This approach is justified since it allows the researcher to use multiple techniques of data collection (triangulations), such as participant observation, interview, field diary records, and document analysis, thus composing a structured field, presupposing the organization and hierarchy of the elements of its content^(^ 9 ^)^. The method also enables the constant interaction between narrator and listener through the hermeneutic circle, proper to its rigor, as a constructive characteristic of the narrative (horizon fusion) that allows the researcher to reach information on culture that is not only rooted in verbal experiences, but rather in actions, with emphasis on the transformations of life^(^ 10 ^-^ 11 ^)^.

For medical anthropology, the disease is not just a biological/body process, but the result of the influence of the cultural context and of the subjective experience indicating that the body is in trouble^(^ 7 ^)^. For the anthropology of masculinities, the theory that observes the positions of power established in gender relations, health and disease are mediators of the practices by which men and women commit themselves to the masculine and feminine places in society and their effects on culture and corporal experience^(^ 8 ^)^.

The research was approved by the Ethics and Research Committee of the University of São Paulo at the School of Nursing of Ribeirão Preto under protocol 054/2013, and all ethical precepts are respected in its processes, as determined by Resolution 466/12 of the National Council of Health. The Informed Consent Form (ICF) was signed by all participants, whose anonymity was assured by replacing their real names with fictitious ones.

Inclusion criteria were: men diagnosed with PC for at least six months under treatment, over 18 years of age, regardless of educational level and socioeconomic status, who self-reported being in physical and mental conditions to narrate their experiences. Among men undergoing therapeutic follow-up at a university hospital located in the countryside of the state of São Paulo, 17 were selected to participate in the study for meeting the pre-established criteria. 

In this study, the data were collected by the first author, individually with each participant through in-depth interviews (at least four meetings lasting two hours, on average) and observation of the research context (such as corporal and sentimental expressions, and subjective facts), which were recorded in a field diary, made at the hospital complex facilities as well as at the places where participants considered it appropriate to report their experience, such as residences, free markets, besides work and leisure places. The records collected are products of the researcher’s experience with the sick during several significant moments in their lives, such as follow-up during consultations and examinations, interaction with family and friends, and socialization with other men. Every meeting was guided by an initial script to assist in data collection; clinical events were compiled from the participants’ medical records, and information on their experiences was evoked by guiding questions such as: What is it like to deal with CP treatments? What has changed in your life? What do you do to take care of yourself? What does it mean to be a man to you? Has your life as a man changed? It is worth mentioning that, according as they expressed their experiences, other questions were formulated for each participant in order to access the nuances of each story and actions presented. After each meeting, the data were compiled and analyzed with the possible reflections recorded in the field diary, and then new questions were raised and deepened during the subsequent meetings. Fieldwork immersion lasted 36 months and only terminated when the goal set was reached and the data began to repeat, indicating saturation, and new meanings were no longer added. 

After data collection, individual narrative syntheses were constructed followed by collective narrative syntheses to better understand the experiences reported. The scripts were then submitted to inductive thematic analysis^(^ 12 ^)^ with integration of similar and particular aspects of the narratives, presented as a thematic narrative synthesis, which is presented in italics as the primary interpretation, literally as they were produced, besides exemplifying the authors’ analysis.

In the fieldwork, it was assumed that the man sick with PC had the experience to be known, that is, the primary understanding. Ethnographic work was focused on this man, and his experience was learned through interviews, observations, field diary records, and reflections, besides time spent with him. The data obtained were then transcribed and codified to become explanatory and this text was analyzed, there being reflection on its parts and on its whole in order to understand the meanings expressed in the script. In the process, the interviewees were consulted again to strengthen the investigation whenever new knowledge gaps emerged and there was need of further explanation to compose their experiences. When doubts were resolved (until that moment), the men’s experience text was submitted to thematic analysis, which through induction, characteristic of its rigor, allowed fusing horizons (union of common sense with scientific knowledge) and constructing, in an explanatory way, the scripts of their experiences through stories narrated, which were presented to the interviewees for validation, thus getting the meanings, that is, a secondary understanding of the facts. However, even after all this process, during reflexivity, if the parts did not fit the whole and the whole did not fit the parts of the text, men were consulted again to clarify doubts and obtain new perspectives that helped learn more about their experiences and structure a comprehensible explanation of their narratives. This coming and going with the sick, with the analysis, with the narratives and with reflexivity was present during all stages of this research, and access was only possible because the hermeneutic circle was adopted as a guide for authors’ actions.

**Figure 1:**
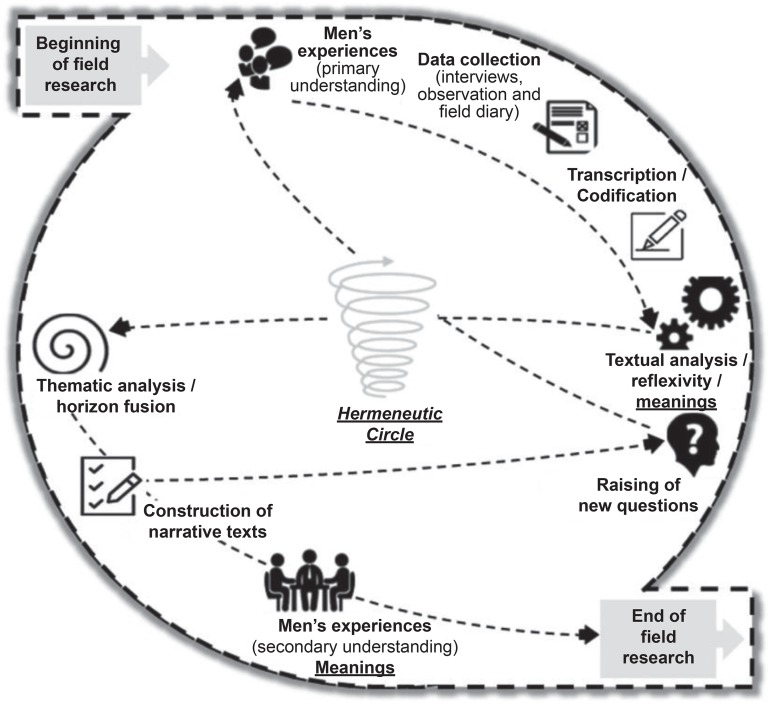
Flow - Summary of the methodological process adopted

Figure 1 summarizes the methodological process structured in this investigation

## Results

The group of participants consisted of 17 men (Fabio; Reinaldo; Antonio; Tony; Alexandre; Thiago; Miguel; Rodrigo; Joaquim; Lucio; Marcos; Mateus; Lázaro; Murilo; Jesus; Vladimir and Cauã). Most of them were married, catholic, retired, living in the city of Ribeirão Preto, São Paulo, Brazil, had their own housing, with incomplete elementary school, and aged between 61 and 81 years. All underwent radical prostatectomy and experienced erectile dysfunction and urinary incontinence. Five of them have been also submitted to radiotherapy sessions and three used complementary therapies with herbs and medicinal teas concomitant to medical treatment

The liminalities experienced by the narrators regarding their experiences in relation to PC and their masculinities demonstrate that masculinity has become an exercise of constant visitation to the concepts of sex and sexuality. Their narratives have been inseparable from these contexts; thus, understanding how they perceive their sexuality and relationships becomes critical to interpreting their experiences. For them, the sexual role that man plays is an undisputed element of his masculinity, and being active occupies the highest point of the culturally established sexual chain, as exemplified in the narrative excerpts: *I am a man because I’ve learned from childhood to be this way (*they were apprehensive and uncomfortable talking about this issue) (Fabio; Reinaldo and Cauã). *My father taught me how to play soccer, ride a horse, drive cattle, drive a car, and do these man’s things; above all, to behave as such, know what to do or not to do, and what I was expected to do within my role. I grew up this way, so I can’t be anything other than what I was taught to be. Man is something natural, so what he has by nature is what makes him to be what he is* (Tony; Alexandre; Jesus and Vladimir). *Man is a kind of leader, more respected than woman, not only in relation to sex but in everything. There is no proper respect when it is said that the family does not count on a man; I think the man is who supports the family; he was born to have control of everything. I don´t have the habit of bragging about myself, but I was an exemplary man. When I talked to people, they respected me, they listened to me because I had my value. I think my serious expression helped, my beard and the way I dressed said a lot about me; I looked like a responsible person* (Antonio; Tony; Alexandre; Lucio; Marcos; Lázaro and Murilo)*; I think a lot about the past, and I remember having lots of women in my life; when I wanted to be with a woman I did so! I was very fond of showing off, I worked out and was always without wearing a t-shirt, everyone looked at me, and it was so good! We feel desired, valued. I had a reputation for being a womanizer until I got married and settled down. Everything was restricted to my wife, we understood each other and we were very happy. I had disposition, manliness enough to be with her and to take care of things in life. At that time I provided my family with what was necessary, and I was respected* (Reinaldo; Tony; Alexandre; Thiago; Miguel; Lucio; Marcos; Thiago).

The perspective on the active sexual life is presented as a social determinant of hegemonic masculinity; however, due to being sick, men were evidenced as fugitives from this function. All the narrators presented sexual dysfunction and translated the meaning of this experience into a sudden illness that stopped their life as men, and although they wanted to restore normality, the biological body did not respond as they would like, as the narratives reflect: *I am a man, but due to being sick I stopped playing my man’s role. This is people’s illusion, no one is 100%, everyone is afraid of something and fails at some point, and no one is totally macho men, because no one is Superman or Batman. When I underwent prostate surgery, I thought the man I used to be would still live. Hmm ... just illusion! People make a point of reminding me that I can’t do anything else because I am a child again. I am not naive and I know that everything you do is judged by others as right and wrong. This is the role we play to live among people; since I’ve undergone surgery I no longer feel like a man, because, as much as I want, my body no longer plays a man’s role. They removed more than the prostate; they took my life as a man. But life is like that, when it does not bring you down it hurts you. Today I am a little stuck to do things, I have no more strength, and I cannot carry weight. Almost everything I used to do I don’t do anymore, so I say I’m different than I was for being full of limits* (Fabio; Reinaldo; Antonio; Tony; Alexandre; Thiago; Miguel; Rodrigo; Joaquim; Lucio; Marcos; Mateus; Lazarus; Murilo; Jesus; Vladimir and Cauã).

Another liminality experienced by the narrators was related to controlling the body, in which the lack of control of the urinary sphincter was symbolized as something that brings inferiority feelings, since they consider themselves different from others because they are constantly wet and with a fetid odor that bothers the people around them, besides keeping them away from work. For being treated differently by family and society, they remain in constant vigilance, as exemplified in the speech: *This operation disgraced my life as a man, because if before my problem was to live with urinary retention, today it is to live with urinary incontinence. The urine that used to be trapped today leaks, and this makes me isolate myself from the world because I am sick and cannot live in society anymore. Even wearing a diaper, a sock, a pad and a piece of fabric to contain the urine, there’s no way, the pants get all wet and I feel ashamed* (Tony; Marcos; Matthew and Jesus). *Using these things is very embarrassing; it hurts the male principle because they are female stuff. Over time, I started realizing that people moved away from me, and at first I didn’t understand what it was, they even fired me because of this; but one day my daughter said it was because the smell of my urine sometimes got too strong, and they didn’t feel well. I felt like a bathroom floor cloth, a real useless person. I’m more comfortable here, currently I know I’m not like other guys, so I isolate myself from the world and do not leave the room for anything, because with this urine issue it is not possible to have a normal life. For me, the world outside home has finished after cancer* (Fabio; Thiago; Miguel; Joaquim; Lucio; Marcos and Lázaro). *Now I’m really in trouble, but no one needs to know it to judge me. Making a comparison with what I was, I’m a new man, I became a child again, because I no longer walk freely, I don’t have sex, I don’t smoke, I don’t drink, I don’t hang out with friends anymore, I urinate in my pants, I wear pads; what will come next? Dying because of cancer is the only thing left, because I don’t even recognize myself. I just wanted to be normal again, just like all the men out there. I want to be manful, have character, work and provide my family with everything* (Reinaldo; Murilo; Jesus; Vladimir and Cauã).

Anchored in sexual dysfunction, anorgasmia was also present as liminality in the narrators’ experiences. Semen was understood as a passport, an identity that every man has and needs to prove during the sexual act. For the narrators, losing ejaculation was like losing a part of themselves, considered an embarrassing process that hurts their identities and hinders their marital and social interaction, described as follows: *I can no longer ejaculate, I became dry and my ejaculation is now internal. These things are very embarrassing, because only the man can show, through ejaculation, that he enjoyed having sex; if the woman wants to fake it, she does so, but the man, he cannot pretend* (Tony; Rodrigo; Joaquim; Lucio; Matthew, Murilo and Vladimir). *For my wife, the semen is some kind of proof that I’m enjoying sex. For the people outside, it’s a kind of stamp, because during sex every man ejaculates for the woman to see it. To escape this rule, I try to disguise, and when I have sex I tell my wife that I ejaculated in a piece of fabric and she does not see it, or we try to have relations in the bathroom and then I pull out my penis and she thinks my semen was taken by water. It kills me slowly! I feel very small because of this! The sperm is my passport, my identity; nowadays, I don’t have it anymore, and then I realized it. I tried to reverse this situation, I went to the doctor and started undergoing treatment; I know it is artificial, because this hardness is not me, it is the medicine. When I take it, one way or another I end up being an artificial man, because I can only have sex with medicine* (Reinaldo; Antonio; Tony; Alexandre; Rodrigo; Joaquim; Lucio; Marcos; Mateus; Lázaro). 

In seeking to return to hegemonic standards, even though they feel like artificial men, the narrators made no effort to balance the biological body, as sex with their partners was shaken by erectile dysfunction and lack of sexual desire, and, even so, they continued with this practice because it was a precept for fulfilling a moral obligation, as illustrated in the excerpt from the narrative: *Looking at me I can see nothing I was but memories. If I don’t get better, it will be not possible for me to live; it is not possible to live in the past. I want to be a real man, I don’t want to be a sick man* (crying) *... I think I’m living in relatively satisfactory health, I have to think like this, wear a mask to go out as if nothing had happened and then no one will judge me for not being a man anymore; and keep on living* (Alexander; Lazarus; Joaquim; Murilo and Jesus). *There are a lot of men who have undergone surgery and are worse than me ... There are guys who even turn into homosexuals! I like women; I just can’t handle them anymore! I’m a half man now. The man who does not feel attraction, does not feel horny, and no longer handle with women, this man cannot be considered a real man! That’s what I have learned since I was a child, but I also know that people only see me as a man if I sleep with someone; otherwise I’m not good for anything. To be honest, after surgery I no longer know which pleasure real sex has, because desire hardly exists. As I am the problem and cannot disappoint her,* *I still have sex, but now it is unwilling sex* (Fabio; Reinaldo; Alexandre; Miguel; Joaquim; Marcos and Mateus).

The excerpt highlights that moral experience also permeates the meanings that men attribute to their masculinity when dealing with body transformations. Whether from the health disease process, from the masculinity or even from the social process, the meaning of transition translated the experience of men in this study and accompanied them in times of identity crisis, where recognizing themselves and keeping on living became a constant process of resignification of reality, as exemplified in the following excerpt: *I changed a lot and there are times when I don’t even know myself anymore. It’s incredible how we transform, the morale that took so long to develop, which we’ve been talking about and constructing since we were little boys, not allowing anyone to touch us but our mother, suddenly goes away, and when we realize, we’re weak. I’m another guy, I’m sick, and it’s the way to live now* (Reinaldo; Alexandre; Thiago; Miguel; Rodrigo; Vladimir and Cauã).

Faced with the rearrangements of liminality and the fear of being punished by moral experience, the narrators reacted on the basis of their culture by implementing compensatory strategies to deal with the transition to the new man, using resignation as a normative consequence to keep on living. The narrative illustrates this aspect and shows that in times of crisis men also resign themselves, which allows them to adapt to the new identity: *I am a different man and the sooner. I have to accept life how it is, I have no other choice. I am a man and these things cannot destabilize me; I am the pillar of my family and if I fall everyone falls, so I have to hold on. We control ourselves and learn to live automatically. I have nowhere to run! Being the man of old times no longer matters ... what matters is what I am going to be from now on* (Fabio; Reinaldo; Marcos; Matthew; Lazarus; Murilo; Jesus; and Vladimir).

## Discussion

Researchers^(^ 13 ^-^ 14 ^)^ state that the body’s disease process undermines the maintenance of hegemonic masculinity, as it presents dilemmas in identity, including subordination, passivity, recognition of emotions, and dependence. Specifically in cancer, men reconfigure their physical and social identity and seek to preserve their family masculinity status as much as possible, but liminality is an inevitable process, as biological imbalance reflects directly on social actions by altering male identity.

With regard to the meanings of living with urinary incontinence, sexual dysfunction and all the transformations that occurred from the treatments for PC, presented in this study, it was evidenced that the experiences of men, even with the advent of the evolution of advanced therapeutic techniques, are still represented by conflicting moments that interfere with the way the sick cope with their lives in the social world. Thus, it is noteworthy that reestablishing the treatment for the biological body without implementing care to reestablish the social body is not enough, since man lives his life in a continuous flow of stories and ruptures, with masculinities connected to this flow.

Faced with the globalizing process that permeates male culture, masculinities never occupy the same position within the patchwork; they are always allocating, modifying and adding new meanings through experiences^(^ 8 ^)^. In this perspective, it is interpreted that the meanings presented in the narratives show that, during disease, men suffer transitions in their masculinity because they cry, suffer, transform the functions of their bodies, feel weak and get sick, resignifying their hegemonic experiences and adopting new identity reconfigurations which bring out multiple masculinities. Among the new configurations adopted by them, it is understood that their bodies occupied subordinate positions in relation to other healthy bodies, which are marginalized in their social relations and allied with regard to establishing their affective relationships.

The narrators’ experience of being sick was shaped by a central masculinity, that is, a hegemonic masculinity moderated by identity domains of a retrospective past to which they belonged before the disease and that today they try to claim, logically reacting to adversities by dealing with them, aiming to take back control of the functions of their bodies. Thus, in this relation, there is a real man of the present and an idealized man of the past, a paradox that for the narrators comes with uncertainties of the future. Faced with this paradox, based on the experiences reported and reflexivity on the data gathered in this study, there was integration of the elaborated meanings and liminality was interpreted as an expressive way assumed by the male cultural acts in the sick man’s experience, and due to this characteristic, it was understood that this narrative has the identity transition as its central meaning.

Transition is an anthropological concept that occurs throughout the various phases of an experience and implies a change or logical reaction to an event^(^ 5 ^)^. It is a concept where the feeling of uncertainty and disturbance of identity is shared, as people transit in a space where they do not identified with one thing or another, or perhaps identify with both at the same time, since the identities do not more easily fit into categories and simply transit^(^ 15 ^)^. Process, perception and disruption are universal characteristics of the transition, which has the ultimate goal of achieving a well-being status^(^ 15 ^-^ 16 ^)^. 

The transition process is dynamic and multiple within an experience and can be classified into four types: situational, health/disease, organizational and developmental^(^ 15 ^,^ 17 ^)^. It is pointed out that this study was focused on the health/disease transition, as it is considered to be more representative of the experience of the man with CP in every process that deals with the new identity. 

When getting sick, men certainly acquires transitional experiences that are confronted by ruptures of some ties that support and strengthen the feelings of security in the hegemonic world, such as those presented in the narratives. The transformation of these ties puts masculinities in a game of disputes that rearrange their identities.

However, in combining the transformations and transitions in an anthropological speech, it should be clarified that the concept governing the transition is not the same as the one governing the transformation, since in culture they do not communicate as synonyms. Transformation converges to a situation in life and transition is the experience related to this situation, that is, the transformation can occur outside a phenomenon and the transition can only occur by experiencing this phenomenon^(^ 18 ^)^. From this perspective, it is possible to say that not every transformation generates a transition, but certainly every transition, whether granted or not, generates a transformation.

As a science, nursing advances with the help of various philosophical approaches, because the profession assumes the perspective of the transitions resulting from the health and disease process, as well as from other life phenomena. Providing assistance to others who deal with changes in their body and well-being is under nurses’ competence^(^ 15 ^,^ 17 ^)^. Therefore, it is pointed out that it is paramount to identify and characterize the transitions that men live in the course of their lives, so that later it will be possible to develop a plan of care individualized to real needs.

Whether from the health disease process, from the masculinity or even from the social process, the meaning of transition translated the experience of men in this study and accompanied them in times of identity crisis, where recognizing themselves and keeping on living became a constant process of resignification of reality. 

From the perspective of common sense, resignation receives a conceptualization equivalent to accepting, allowing or agreeing with something. However, under the lens of anthropology, resignation is a sense of tension that individuals experience between fighting for something or simply giving up^(^ 19 ^)^. Faced with this situation, they become powerless, as they no longer have the necessary elements to influence their future and reach a possible or visible exit. Thus, being resigned is an acceptance process when experiencing an adverse situation even when it requires changes that are only possible at a great price or risk, and these changes are not necessarily desired by those who assume them^(^ 20 ^)^.

In the context investigated, it was evidenced that male identities resigned to the experience of the disease, as they did not connect with the precepts of hegemonic masculinity, despite recognizing it as a pattern to be followed. By resigning, men felt oppressed by their own feelings and had difficulty managing their male identities.

Faced with a disease such as cancer, recognized for being stigmatized, mutilating, which generates uncertainties and responsible for triggering so many changes in the biological and social body, men stand “between a rock and a hard place,” that is, they either accepted the disease and incorporated a new identity or remained stable and suffered the consequences of getting sick. Amid this “crossroads,” resigning was the narrators’ way of continuing to survive in some way, even if anchored in memories of their past, since the present is challenging and the future is uncertain in the new male identity that lives in their bodies.

The experiences presented in this research are relevant to the enhancement of knowledge of the subject, as they highlight the actions and subjectivities present during the body and identity transitions of the sick, in addition to elucidating the moral dilemmas faced in the defense of their masculinities. These are significant evidence for the prescription of complete nursing care and, consequently, increased quality of life.

However, because it is a research that evidenced local masculinities, it is noteworthy that it has limitations, since it summarizes the experiences of men inserted in a given culture, and masculinities influenced by a multiple culture. Other masculinities coexist at regional and global levels, which relate to those presented, which would broaden the understanding of the phenomenon studied.

## Conclusion

The narratives allowed evidencing that PC provides the sick with liminalities in the experiences in dealing with sexual dysfunction, urinary incontinence, identity crisis, loss of hegemonic masculinity, among others. Faced with these complications, the man who was strong and virile became a dependent and isolated man who logically reacts to the disease by transforming into a new man. From this perspective, it is concluded that man is able to go through multiple masculinities in the search for preserving his male status.

The masculinities presented enhance and deepen the knowledge disclosed in the literature on the experiences of the sick with PC, contribute to strengthening treatments, education and research directed at this population, besides helping nurses in their care actions when dealing with the sick. However, the need for future studies that emphasize the analytical character of the experience of other subjects involved in the care process, such as family members, caregivers and health professionals, is emphasized.
